# Distinctive DNA mismatch repair and APC rare variants in African Americans with colorectal neoplasia

**DOI:** 10.18632/oncotarget.21557

**Published:** 2017-10-07

**Authors:** Hassan Ashktorab, Hamed Azimi, Sudhir Varma, Payaam Tavakoli, Michael L. Nickerson, Hassan Brim

**Affiliations:** ^1^ Department of Medicine and Cancer Center, Washington, DC, USA; ^2^ Department of Pathology, Howard University College of Medicine, Washington, DC, USA; ^3^ Hithru LLC, Silver Spring, MD, USA; ^4^ Laboratory of Translational Genomics, National Cancer Institute, Bethesda, MD, USA

**Keywords:** targeted exome sequencing, colon, African Americans

## Abstract

**Purpose:**

African Americans have a higher incidence and mortality from colorectal cancer. This disparity might be due, in part, to the type of mutations in driver genes. In this study, we examined alterations specific to *APC*, *MSH3*, and *MSH6* genes using targeted exome sequencing to determine distinctive variants in the course of neoplastic transformation.

**Experimental Design:**

A total of 140 African American colon samples (30 normal, 21 adenomas, 33 advanced adenomas and 56 cancers) were used as our discovery set on an Ion Torrent platform. A 36 samples subset was resequenced on an Illumina platform for variants’ validation. Bioinformatics analyses were performed and novel validated variants are reported.

**Results:**

Two novel *MSH6* variants were validated and mapped to the MutS-V region near the MSH2 binding site. For *MSH3,* 4 known variants were validated and were located in exon 10 (3 non-synonymous) and exon 18 (1 synonymous). As for *APC*, 20 variants were validated with 4 novel variants: 3 stopgain and 1 non-synonymous. These variants mapped prior to and on the Armadillo repeats region, to the 15-amino acid repeat region, and to the 20-amino acid repeats region, respectively.

**Conclusion:**

We defined novel variants that target DNA mismatch repair and *APC* genes in African Americans with colorectal lesions. A greater frequency of variants in genes encoding DNA mismatch repair functions and *APC* likely plays major roles in colorectal cancer initiation and higher incidence of the disease in African Americans.

## INTRODUCTION

Colorectal cancer (CRC) is the third most commonly diagnosed malignancy in the World [1]. Despite advances in early detection and therapies, CRC still has a lethal outcome in nearly 40% of all diagnosed cases [2, 3]. CRC is an important contributor to cancer mortality [4]. Alterations in oncogenes, tumor suppressors, and DNA mismatch repair (MMR) genes lead to CRC development [5, 6]. The significant clinical heterogeneity within a given pathologic stage reflects the underlying molecular heterogeneity in CRC pathogenesis [3].

Molecularly, CRCs are categorized into those with microsatellite instability (MSI) which are primarily proximal and frequently associate with the CpG island methylator phenotype (CIMP) and hypermutation, and those that are microsatellite stable (MSS) but are chromosomally unstable [3, 7, 8]. MSI characterizes 10–15% of sporadic CRCs and has been related to a better patient prognosis compared with MSS colorectal cancer [7–12].

Tumors that demonstrate high MSI (MSI-H) as a result of alteration/loss of mismatch repair (MMR) protein(s) are referred to as *MMR*-deficient. *MMR*-proficient tumors include MSS and likely MSI-low tumors (MSI-L). The determination of *MMR* status is important for diagnosis and treatment.

DNA mismatch repair system is composed of 6 proteins (*MLH1, MSH2, MSH3, MSH6, PMS1* and *PMS2*) whose function is to correct base-base mispairs introduced into short tandem repeats, termed microsatellites, during DNA synthesis [9, 13, 14]. We and others have previously reported the primary involvement of *MLH1* and *MSH2* alterations in MSI-H phenotype occurrence. However, alterations of *MSH3* and *MSH*6 were also cited with proximal tumors that are poorly differentiated and mucine positive [14, 15].

*APC* is one of the key tumor suppressor genes (TSG) associated with early colon carcinogenesis [16, 17] in both familial adenomatous polyposis coli (FAP) and FAP-like sporadic CRCs [18]. Recent studies have shown mutations of *APC* in many cancers including CRC [7, 11, 17, 19–21]. *APC’s* impact on downstream effectors such as β-catenin, GSK and AXIN has been established in previous studies [17, 19, 21–24]. Altered WNT pathway signaling plays a significant role in CRC development and genetic variation in *APC* has been shown to correlate with increased predisposition to CRC [11].

We recently analyzed 12 pairs of African American CRC tumors and their matched normal tissue using whole exome sequencing and established a panel of novel variants that are potentially useful for better subtyping of CRC in this population [11]. We have reported *APC, MSH3,* and *MSH6* among the top target genes for mutations. As such we further analyzed them through targeted sequencing in a larger sample size.

In this study, we examined *APC, MSH3,* and *MSH6* sequence alterations and copy number variations (CNV) using targeted exome sequencing (TES) to determine specifics of these genes variants’ profiles in African American patients.

## RESULTS

### Clinico-pathological characteristics of patients

**Figure 1:**
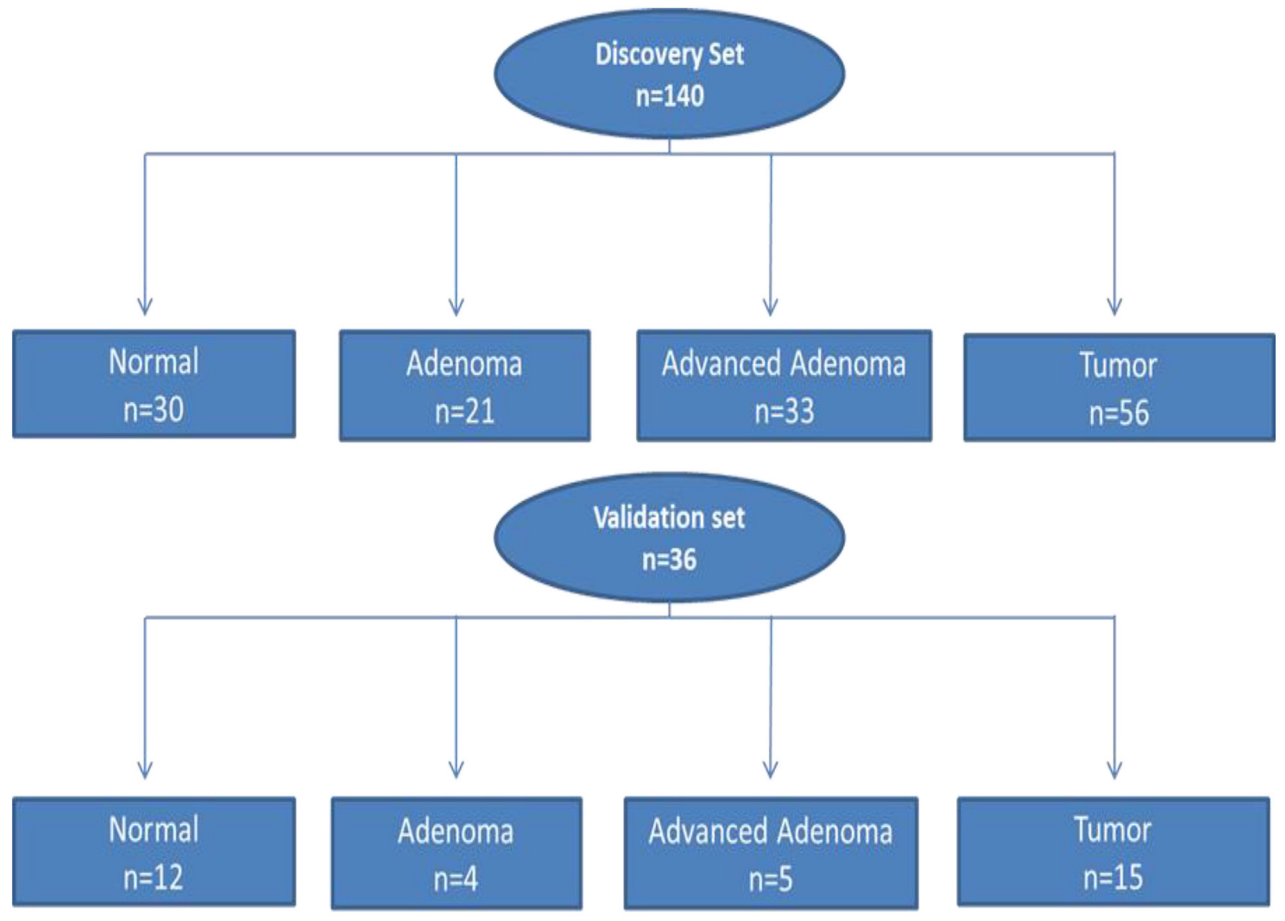
Flow chart of patient selection for both Discovery and Validation sets for somatic variant analysis Discovery set: 140 samples (n=123 patients) and Validation set: 36 samples (n=26 patients).

A: Discovery set (Ion Torrent sequencing [Thermo Fisher Scientific; Waltham, MA]): The study (discovery) set consisted of 123 patients (140 samples) (Figure 1 and Supplementary Table 1). Of these patients, 52 (43%) were females and 69 (57%) were males (for 2 samples, gender is missing). The age range of patients was 24 to 95 years, with a median of 62 years. With regards to cancer stage, 11% (6/56) were stage I, 34% (19/56) were stage II, 29% (16/56) were stage III, and 4% (2/56) were stage IV [23% (13/56) had no staging data].

B: Validation set (Illumina sequencing; San Diego, CA) was performed on a subset of the study group. This subset (Validation set) consisted of 36 samples from 26 patients. The main goal of this second sequencing is to rule-out technical sequencing artifacts and eliminate false variants. There were 11 (42%) females and 15 (58%) males. With regard to cancer stage, 60% (n = 9/15) were stage II, 26% (n = 4/15) were stage III and 13% (2/15) were stage IV. The age range was from 41 to 88 with a median age of 55 years (Table 1).

**Table 1:** Clinico-pathologic characteristics of the Validation set patients (n=26, 36 samples) with previously described and novel variants

Sample ID	Age	Sex	Type of Tissue	Location	TNM	STAGING	*APC* Mut	MSH6
**CC1018N**	84	F	N	RIGHT	N	NA	-	-
**CC1018**	84	F	T	LEFT	T3N1b	II	-	-
**CC1024**	73	F	T	RIGHT	T3N1MX	III	+	+
**CC1028**	42	M	T	LEFT	T3N0MX	II	+	-
**CC1028N**	42	M	N	RIGHT	N	NA	-	-
**CC1029**	51	F	T	RIGHT	T2N0MX	II	+	+
**CC1029N**	51	F	N	LEFT	N	NA	-	-
**CC1036**	63	M	T	LEFT	T3N2M1	IV	+	-
**CC1036N**	63	M	N	RIGHT	N	NA	-	+
**CC1038**	54	M	T	LEFT	T3N0Mx	II	+	-
**CC1038N**	54	M	N	RIGHT	N	NA	-	-
**CC1053**	50	F	T	RIGHT	T3N0M0	II	+	-
**CC1053N**	50	F	N	LEFT	N	NA	-	-
**CC1054**	53	M	T	RIGHT	T3N0M0	II	+	+
**CC1054N**	53	M	N	LEFT	N	NA	-	-
**CC1055**	79	F	AA	RIGHT	AA	NA	+	
**CC1056**	66	M	T	LEFT	T1N1MX	III	+	-
**CC1056N**	66	M	N	RIGHT	N	NA	-	-
**CC1057**	88	M	T	LEFT	T3N2M0	III	+	-
**CC1057N**	88	M	N	RIGHT	N	NA	-	-
**CC1059**	60	F	T	RIGHT	T3N1MX	II	+	+
**CC1060**	53	F	T	LEFT	T3N2M1	IV	+	
**CC1060N**	53	F	N	RIGHT	N	NA	-	+
**CC1061N**	63	M	N	LEFT	N	NA	+	-
**CC1065**	41	M	T	LEFT	T3N0M0	II	+	-
**CC1109**	62	F	A	RIGHT	A	NA	+	+
**CC1258**	52	M	T	RIGHT	T3N1bMx	III	+	-
**CC1386**	70	M	T	LEFT	T3N0Mx	II	-	-
**CC1621N**	49	M	N	RIGHT	N	NA	+	-
**CC1680**	75	F	AA	RIGHT	AA	NA	-	-
**CC1681**	75	M	AA	LEFT	AA	NA	+	-
**CC1682**	71	M	AA	RIGHT	AA	NA	+	-
**CC1683**	45	M	A	RIGHT	AA	NA	+	-
**CC1698**	54	M	A	RIGHT	A	NA	+	-
**CC1720**	70	F	A	RIGHT	A	NA	+	-
**CC1721**	54	M	A	Missing	A	NA	+	+

N= Normal, T = CRC, A = Adenoma, AA = Advanced Adenoma, - =No variant. NA=Not Applicable

### MMR genes

*MSH3*: We detected 315 *MSH3* variants in the Discovery set, of which 298 were novel. Among these, 273 were non-synonymous, 23 were stopgain, and 2 were frameshift variants. Seventy variants altered the MutS_V domain, 21 altered the MutS_III domain, 37 altered the MutS_II domain and 32 altered the MutS_I domain (S.1).

**Figure 2:**
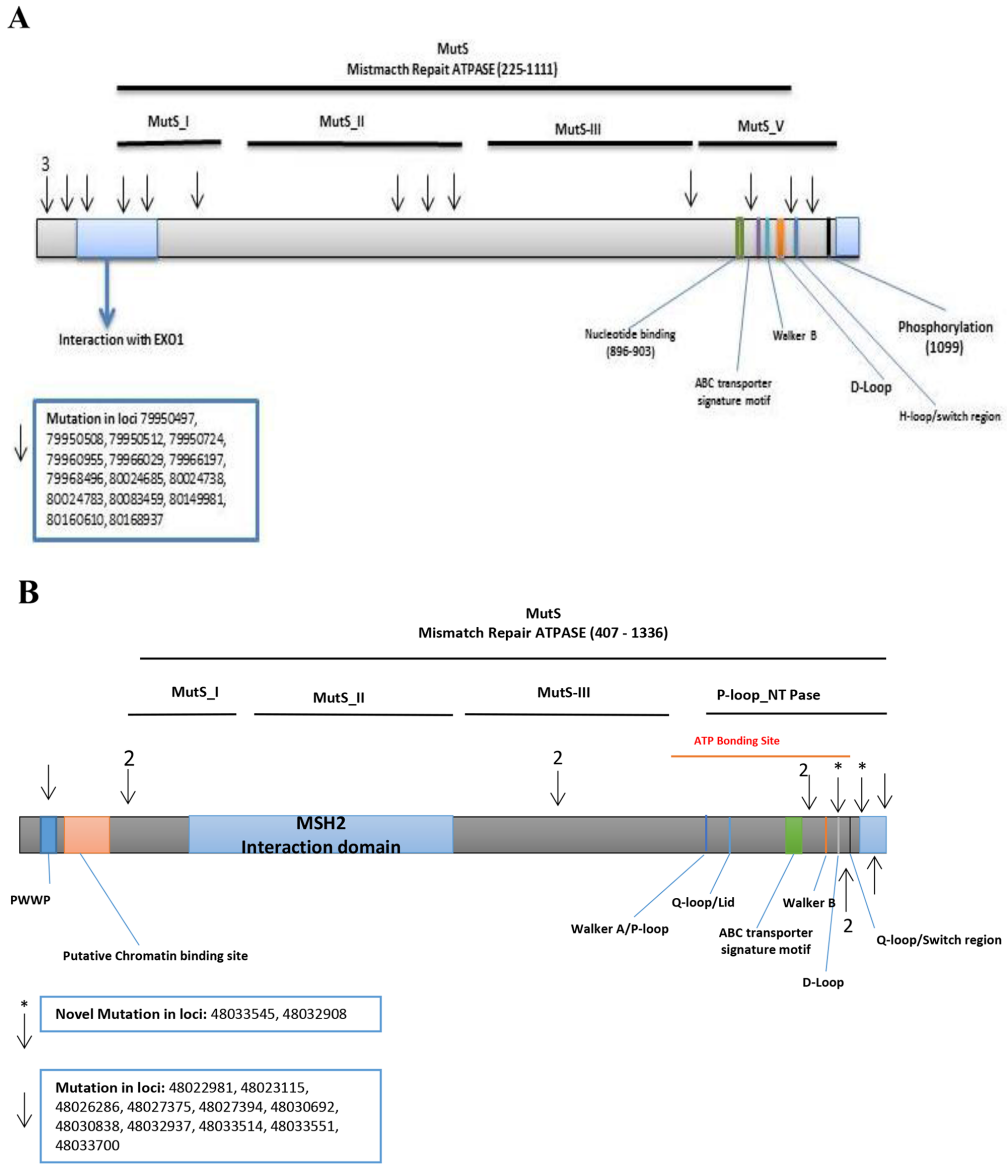
Distribution of confirmed and novel variants in proteins encoded by target genes **(A)** MSH3 **(B)** MSH6, (Arrows show confirmed and asterisks the novel variants, respectively).

Of the above variants, 14 previously identified variants were confirmed by Validation set using Illumina sequencing (Table 2). Six of these variants were non-synonymous altering exons 10, 21, and 23. Two were synonymous changes in exon 4 and 18. Variant at locus chr5:79966029 (HG19 reference) with a G to A change, had a frequency of 0.23 (7/30, 1 homozygous and 6 heterozygous) in normal, 0.52 (11/21, all heterozygous) in adenoma, 0.21 (7/33, 2 homozygous and 5 heterozygous) in advanced adenoma, and 0.29 (16/56, all heterozygous) in CRCs. Variant at locus chr5:80083459 with a G to A change, had a frequency of 0.03 (1/30, heterozygous) in normal, 0.06 (2/33, both heterozygous) in advanced adenoma, and 0.02 (1/56, heterozygous) in CRCs. The other variants were flanking intronic. The variants were mapped to the MSH3-MSH2-MSH6 region with 4 prior to EXO1, 3 in EXO1, 1 in MutS_I, 3 in MutS_II, 1 in MutS_III, and 3 in MutS_V domain (Figure 2A).

**Table 2:** Number of samples with confirmed variants in the targeted gene panel

						Number of samples							
Loci	Ref	Var	Gene	Type	Novel	AA-Normal Het (30)	AA-Normal Hom (30)	AA-Adenoma Het (21)	AA-Adenoma Hom (21)	AA-Ad. Adenoma Het (33)	AA-Ad. Adenoma Hom (33)	AA-CRC Het (56)	AA-CRC Hom (56)
112043384	T	G	APC	Intronic	0	6	0	3	0	7	1	12	0
112103015	C	A	APC	Stopgain	0	0	0	0	0	0	0	0	0
112116592	C	T	APC	Stopgain	0	0	0	0	0	0	0	2	0
112128191	C	T	APC	Stopgain	0	0	0	1	0	2	0	2	0
112136947	A	T	APC	Intronic	0	3	0	2	0	3	0	3	0
112151261	C	T	APC	Stopgain	0	0	0	0	0	1	0	1	0
112154942	C	T	APC	Stopgain	0	0	0	1	0	1	0	1	0
112154980	T	A	APC	Stopgain	1	0	0	1	0	0	0	0	0
112157658	G	T	APC	Stopgain	1	0	0	1	0	0	0	0	0
112162854	T	C	APC	Synonymous SNV	0	11	1	7	2	14	2	17	6
112164561	G	A	APC	Synonymous SNV	0	16	5	14	6	20	7	27	16
112173553	T	G	APC	Synonymous SNV	0	1	0	1	0	0	0	0	0
112173899	C	T	APC	Non-synonymous SNV	0	1	0	1	0	2	0	3	0
112174096	C	A	APC	Stopgain	0	0	0	0	0	1	0	0	0
112174763	A	T	APC	Stopgain	1	0	0	0	0	0	0	1	0
112175023	A	G	APC	Synonymous SNV	0	3	0	2	0	4	0	3	0
112175030	G	A	APC	Non-synonymous SNV	0	1	0	0	0	0	0	2	0
112175069	C	T	APC	Stopgain	0	0	0	1	0	0	0	2	0
112175207	G	T	APC	Stopgain	0	0	0	0	0	0	0	2	0
112175399	A	T	APC	Stopgain	0	0	0	0	0	2	0	1	0
112175576	C	T	APC	Stopgain	0	0	0	0	0	3	0	0	0
112175639	C	T	APC	Stopgain	0	0	0	2	0	4	0	2	0
112175770	G	A	APC	Synonymous SNV	0	17	5	13	7	20	8	26	14
112176325	G	A	APC	Synonymous SNV	0	19	4	14	6	21	6	26	17
112176541	C	G	APC	Synonymous SNV	0	1	0	1	0	0	0	2	0
112176559	T	G	APC	Synonymous SNV	0	18	4	13	7	19	8	26	17
112176756	T	A	APC	Non-synonymous SNV	0	3	26	4	17	8	24	4	50
112176918	G	C	APC	Non-synonymous SNV	1	0	0	0	0	1	0	0	0
112177171	G	A	APC	Synonymous SNV	0	16	5	14	6	21	7	31	15
112178492	C	T	APC	Synonymous SNV	0	0	0	0	0	1	0	1	0
112178795	G	A	APC	Non-synonymous SNV	0	1	0	0	0	0	0	2	0
112178995	A	G	APC	Synonymous SNV	0	7	0	3	0	6	0	6	2
112179909	C	A	APC	Intronic	0	9	1	9	1	12	0	20	3
79950497	C	T	DHFR, MSH3	Intronic	0	6	4	6	2	3	2	14	5
79950508	C	T	DHFR, MSH3	Intronic	0	4	0	1	0	3	0	5	0
79950512	A	G	DHFR, MSH3	Intronic	0	5	14	1	8	3	8	13	26
79960955	G	A	MSH3	Intronic	0	11	5	10	4	16	1	24	7
79966029	G	A	MSH3	Synonymous SNV	0	6	1	11	0	5	2	16	0
79966197	G	A	MSH3	Intronic	0	11	5	11	4	19	1	26	5
79968496	C	T	MSH3	Intronic	0	5	3	3	3	11	1	13	3
80024685	C	A	MSH3	Non-synonymous SNV	0	0	0	2	0	0	0	0	0
80024738	A	G	MSH3	Non-synonymous SNV	0	0	0	0	0	2	0	0	0
80024783	G	A	MSH3	Non-synonymous SNV	0	1	0	0	0	5	0	2	0
80083459	G	A	MSH3	Synonymous SNV	0	1	0	0	0	2	0	1	0
80149981	A	G	MSH3	Non-synonymous SNV	0	2	27	4	16	7	23	5	48
80160610	T	A	MSH3	Intronic	0	5	2	4	0	4	1	12	1
80168937	G	A	MSH3	Non-synonymous SNV	0	11	15	10	8	19	10	22	30
48022981	G	T	MSH6	Intronic	0	10	3	12	1	11	1	23	4
48023115	T	C	MSH6	Synonymous SNV	0	9	1	5	1	7	1	14	1
48026286	C	T	MSH6	Synonymous SNV	0	4	0	2	0	4	0	7	0
48027375	T	C	MSH6	Synonymous SNV	0	4	0	2	0	7	0	5	1
48030692	T	A	MSH6	Synonymous SNV	0	0	0	0	0	1	0	0	0
48030838	A	T	MSH6	Intronic	0	10	2	12	0	8	1	17	2
48032908	A	G	MSH6	Intronic	1	1	0	1	0	0	0	2	0
48032937	T	C	MSH6	Intronic	0	4	25	4	17	9	21	12	40
48033514	T	C	MSH6	Intronic	0	0	0	2	0	2	0	1	0
48033545	A	C	MSH6	Intronic	1	0	0	0	0	0	0	1	0
48033551	C	G	MSH6	Intronic	0	4	20	3	11	8	9	13	34
48033700	G	A	MSH6	Non-synonymous SNV	0	1	0	2	0	0	0	2	0

*MSH6*: We detected 434 variants in the Discovery set, of which 396 were novel (Supplementary table 2). Of these, 371 were non-synonymous, 22 were stopgain and 3 were frameshifts. Seventy-six variants altered the MutS_V domain, 29 altered the MutS_IV domain, 31 altered the MutS_III domain, 52 altered the MutS_II domain and 24 altered the MutS_I domain (S.1).

The Validation set sequencing confirmed 12 variants (by Illumina). Two of these were novel (Table 2). The A>G and A>C variants are flanking the MutS_V domain and the MSH2 binding site coding regions, respectively. An A>C intronic variant (IVS8-45) at locus chr2:48033545 was observed in 1 CRC sample with a frequency of 0.02 (1/56, heterozygous). The A>G variant at locus chr2:48032908 was intronic (IVS7-50) and was observed with a frequency of 0.03 (1/30, heterozygous) in normal, 0.05 (1/21, heterozygous) in adenoma, and 0.04 (2/56, both heterozygous) in CRC (Table 2). One variant was mapped in the PWWP domain, 2 prior to the MutS_I, 1 in MutS_III, and 8 in P-loop_NTPase (Figure 2B).

### *APC* variants

**Figure 3:**
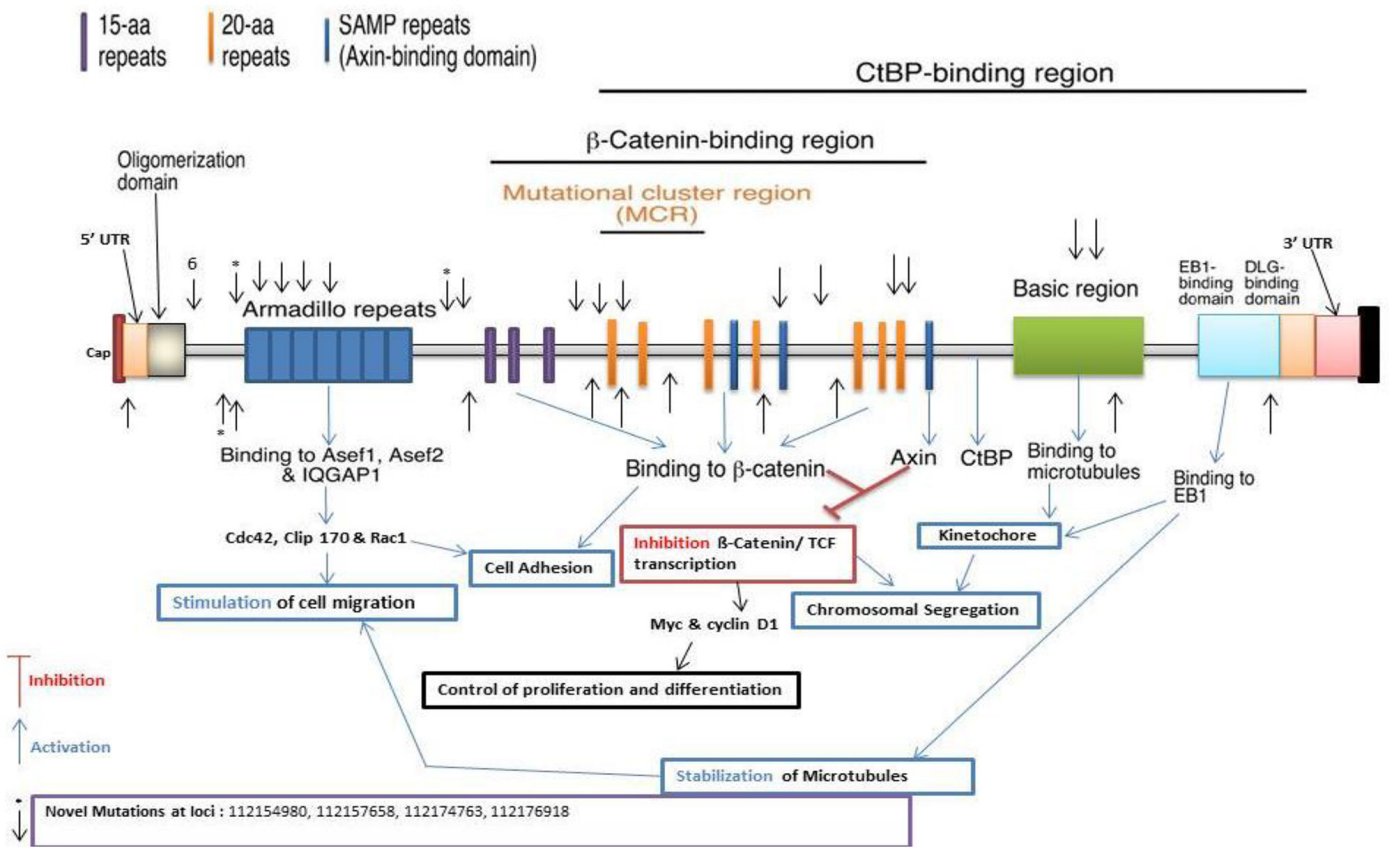
Distribution of *APC* confirmed and novel variants, (Arrows show confirmed and asterisks the novel variants, respectively)

We detected 944 variants in the Discovery set, of which 822 were novel. The Validation set sequencing led to the confirmation of 33 variants of which 4 were novel. The variant at locus chr5:112176918 with a G to C change, had a frequency of 0.03 (1/33, heterozygous) in advanced adenoma. The variant at locus chr5:112174763 with an A to T change, had a frequency of 0.02 (1/56, heterozygous) in CRCs. The variant at locus chr5:112154980 with a T to A change, had a frequency of 0.05 (1/21, heterozygous) in adenoma. The variant at locus chr5:112157658 with a G to T change, had a frequency of 0.05 (1/21, heterozygous) in adenoma. One variant was mapped in the 5’ UTR, 9 prior to ARM, 4 on the ARM domain, 3 prior to β-catenin interacting region, 12 in the β-catenin interacting region, 3 in the basic region, and 1 in the 3' UTR (Table 2 and Figure 3).

We analyzed copy number variation (CNV) and loss of heterozygosity (LOH) in 16 matched tumor and normal samples in a blinded fashion by examining the sequencing read counts of 8978 variants arranged by patient, gene, and genomic location, as previously described [25, 26]. An NGS read counts imbalance of ≥0.6 (variant reads / reference + variant reads) across a minimum of 3 consecutive variants was used as criteria for a CNV/LOH alteration.

Fifteen of 16 pairs of samples were successfully analyzed, and 39 CNV/LOH were detected with an average of 1.6 alterations per tumor. Potential germline CNV/LOH (30/39, 77%) were observed, reflecting allele imbalance in both normal and tumor tissue in 11/15 (73%) pairs, that may be also attributed to PCR bias. Somatic CNV/LOH (9/39, 23%), heterozygous in normal tissue and homozygous in tumors, were observed in 6 pairs of tissues (40%). Two pairs of samples showed evidence of both germline and somatic CNV/LOH in different genes. *MSH6* was most frequently altered, in 11 pairs (73%) by 8 germline and 3 somatic alterations; *APC* was altered in 8 pairs (53%) by 3 germline and 5 somatic alterations, and *MSH3* in 5 pairs (33%) by 4 germline and 1 somatic alteration. CNV/LOH were examined in an additional 93 tumors using the criteria above and 83 samples (89%) were successfully analyzed to detect 117 alterations in 71/83 samples (86%) with an average of 1.7 alterations per sample for the 3 analyzed genes. Similar to the frequencies of gene sequence alterations observed in the matched T-N pairs, *MSH6* was most frequently altered in 48 samples (68%), *APC* in 41 (58%), and *MSH3* in 28 (39%). In total, the T-N pairs and the additional samples exhibited 156 CNV/LOH in 86/98 (88%) samples which altered *MSH6* in 59/98 cases (60%), *APC* in 49 cases (50%), and *MSH3* in 33 cases (34%).

## DISCUSSION

Targeted exome sequencing can lead to the discovery of new targets for prevention, treatment, and diagnostic value through the definition of specific driver mutations, especially in African Americans, a population at high risk of CRC that generally presents aggressive and advanced tumors at diagnosis. In this study, we determined the frequencies of novel and known variants in neoplastic tissues in African Americans with CRC. We examined *APC*, *MSH3* and *MSH6* variants using TES and in-silico analysis of novel variants.

Platform usage in NGS is known to contribute to sequencing outcome and artifacts. We recently reported NGS outcome differences within and across different sequencing platforms which mandate the confirmation of predicted NGS variants [27].

While the frequency of many *MSH3* variants was high, the level of homozygosity is low, which likely points to a deactivation of *MSH3* through different variants. Indeed, several samples displayed two or more *MSH3* variants at once (Table 2). It is noteworthy that many of the validated variants were present in normal matched specimens as well. These specimens were isolated from adjacent areas of the colonic lesions, and as such might correspond to a field effect of the neoplastic lesion, but are most likely germline. One limitation of confirming the somatic or germline nature of these variants is the lack of peripheral blood sample DNA.

For *MSH6*, two novel variants (Table 2) were flanking the region coding for the MutS-V domain and the MSH2 binding site, respectively. One variant was mapped in the PWWP domain, 2 prior to the MutS_I domain, 1 in MutS_III domain, and 8 in P-loop_NTPase domain (Figure 2B).

The majority of sequencing data deposited in public databases are from Caucasians. Minorities are generally underrepresented in such databases including TCGA and ClinVar [28]. This might be one of the reasons of the novelty of some of the variants we describe here. As such, there is a need to characterize the variant profile of all MMR genes associated with genomic instability in ethnically-diverse CRC patients.

*APC* mutations were associated with increased predisposition to colorectal neoplasia [17, 29]. While none of the *APC* variants presented here was homozygous, most specimens displayed 2 or more variants of which the cumulative effect is likely to impair APC function (Table 2). The confirmed *APC* variants are likely to lead to major changes in the activity of the APC protein since they are located in exons 5 and 15. Since stopgain and frameshift variants have major effects on APC protein structure, they will have drastic effects on the protein functions and involved pathways [11, 30, 31]. We report here 4 novel variants; three stopgain and one non-synonymous. One variant was mapped prior to the Armadillo repeat, one to the Armadillio repeat, one to the 15-amino acid repeat region, and one at the 20-amino acid repeat region (Figure 3). The variant observed in the 15-amino acid repeat (15R) region of the APC protein will affect the binding of β-catenin to the 15R of APC that is necessary and essential to target β-catenin for degradation. The first 15R displays the highest affinity for β-catenin in the 15R-20R module. Guda et al. recently reported 4 APC variants similar to ours [32, 33]. Sixteen confirmed *APC* previously described variants are described in our earlier CRC whole exome study [10, 11].

It is worth noting that the analyzed genes act as Tumor Suppressor Genes (TSGs), and as such their variants will only be effective if they lead to a complete loss of function. Our CNV analysis revealed that the 3 genes displayed a high level of LOH in favor of variant alleles. Indeed, *MSH6* displayed the highest level of LOH (60%) in the analyzed samples, followed by the *APC* (50%) and *MSH3* (34%). It is also important to note that some of the detected variants were found in normal tissue samples as well. The heterozygous presence of such variants in normal matched colon tissue samples may contribute to a higher genetic predisposition to CRC in AAs as well as early age onset of the disease in this population.

We understand that CRC incidence in African Americans has many other confounding factors, like utilization, access, and equality of treatment, as well as other socio-epidemiological factors that play a strong role in the development and progression of CRC. Our data, however, shows that this population has many novel variants of potentially deleterious nature that might account for part of the clinical phenotype. Indeed, the in-silica analysis showed that the novel variants have far-reaching consequences. The detected *MSH3* and *MSH6* variants seem to disrupt the whole DNA MMR system which will increase the rate of mutation within the whole genome while the variants within the *APC* gene will impact major pathways, including the WNT pathway

We were among the first to report on the MSI status in African Americans that was predominantly linked to *MLH1* and *MSH2* genes’ alterations. We here report the presence of distinctive variants in other DNA MMR genes that might play a role in the MSI status as well [10]. More importantly, alterations within the *MSH3* gene are associated with the EMAST phenotype characterized by instability in tetranucleotide repeats and poor prognosis, even in MSI-H background patients. This phenotype is more prevalent in African Americans [34, 35]. Studies showed that MSI-H patients with EMAST colorectal cancers show diminished prognosis compared to patients without an EMAST phenotype. Since the distribution of EMAST relates to MSH3 gene mutations and it is more prevalent in African Americans this is potentially a reason why some African American MSI-H CRC patients have poor prognosis [34-36].

In conclusion, we defined novel deleterious variants for *APC*, *MSH3* and *MSH6* genes that might serve as novel markers for neoplastic lesions subtyping and CRC risk assessment in African Americans. These and other known variants might serve as targets for personalized and targeted treatment in the future.

## MATERIALS AND METHODS

### Patients and samples

A total of 140 colon samples (Supplementary Table 1), including 30 normal tissues, 21 adenomas, 33 advanced adenomas, and 56 cancers collected from 123 African American patients were used to establish variant profiles by targeted exome sequencing, using a Discovery set of patients analyzed using an Ion Torrent sequencing platform (Figure 1). Patients’ DNA was quantitated using PicoGreen double-stranded DNA binding assay (Thermo-Fisher Scientific, Waltham, MA). DNA metrics were used to confirm the quality of the library preps. Only samples that passed quality control for the NGS, as a part of standard NGS library preparation, were further processed.

To discriminate technical artifacts from real variants, we used a second sequencing platform (Illumina) in a Validation set consisting of 36 samples from 26 patients including 12 normal tissue samples, 4 adenomas, 5 advanced adenomas and 15 cancers. The Validation set samples are a subset of the Discovery set (n=140; Figure 1).

### Discovery set sequencing

A targeted, multiplex PCR primer panel was designed using the custom Ion Ampliseq Designer v1.2 (Thermo Fisher Scientific). The primer panel covered 56.9 kb and included the coding regions of *APC, MSH3* and *MSH6* genes, with an average coverage of 96.9% of the protein coding regions and splice junctions. The panel was designed to amplify PCR products appropriate for use with DNA from formalin-fixed paraffin embedded (FFPE) tissue with an average amplicon size of 150 base pairs (bp). Sample DNA (20 ng/primer pool) was amplified using the primer panel, and libraries were prepared using the Ion Ampliseq Library Preparation kit following the manufacturer’s protocol (Thermo-Fisher Scientific, Grand Island, NY). Individual samples were barcoded, pooled, and sequenced on an Ion Torrent Proton Sequencer using the Ion PI Template OT2 200v3 and Ion PI Sequencing 200v2 kits per manufacturer’s instructions. Raw sequencing reads were filtered for high-quality reads, and the adaptors were removed using the Ion Torrent Suite 4.0.4, then reads were aligned to the hg19 reference sequence by TMAP (https://github.com/iontorrent/TS/tree/master/Analysis/TMAP) using default parameters. Resulting BAM files were processed through an in-house quality control (QC) filter and coverage analysis pipeline. BAM files were aligned using GATK LeftAlignIndels module. Amplicon primers were trimmed from aligned reads by Torrent Suite. Variant calls were made by Torrent Variant Caller 4.0 (http://mendel.iontorrent.com/ion-docs/Torrent-Variant-Caller-Plugin.html).

### Validation set sequencing

Details regarding DNA quantification and quality assessment, MiSeq platform sequencing (Illumina, San Diego, CA), SNV and indel detection, and assessment of copy number alterations were performed as previously described [10-12, 37].

### Copy number variation and loss of heterozygosity

Variants were organized by genomic coordinates and used to assess regions likely to be altered by a CNV selected from a minimum of 3 grouped or neighboring variants showing allelic imbalance. Cancer and normal sample sequencing data were analyzed in a blinded fashion as described previously [25, 26]. In optimal cases, multiple consecutive variants showed read count imbalance including ≥1 novel variant not in dbSNP likely to be somatic or a rare germline variant.

### Bioinformatics

#### Comparison of African American CRC data to public databases

We used R software (version 2.15.2, http://www.r-project.org/) to compare the variants in the normal and tumor samples with those in the 1000Genomes database, which represents a nominally noncancerous population and dbSNP database. All samples displayed more or less an equal number of single nucleotide variants (SNVs) in their tumors compared with their matched normal samples. In addition, we compared our results with the recently published African American CRC Whole Exome study by Guda et al [32].

An average sequencing depth of >1000x was achieved and >98% of targeted bases (coding and within 5 bp of intron-exon boundaries) were examined by >10 reads required for variant identification. Variants were annotated using ANNOVAR [38] and filtered using the 1000 Genomes database, which represents a nominally noncancerous population, and dbSNP build 138. In addition, variants were filtered using the COSMIC database, a database of cancer somatic mutations. Variants present in any of those three datasets were marked as non-novel. All samples displayed more or less an equal number of SNVs in their tumors compared with their matched normal samples.

## SUPPLEMENTARY MATERIALS FIGURES AND TABLES






