# Supplementary material for: Distinctive DNA mismatch repair and APC rare variants in African Americans with colorectal neoplasia
Source: Oncotarget. 2017 Oct 7;8(59):99966–77. doi: 10.18632/oncotarget.21557 (PMC5725144; doi:10.18632/oncotarget.21557)
Supplement: Supplementary file 1 [file oncotarget-08-99966-s001.pdf]

Distinctive DNA mismatch repair and APC rare variants in African Americans with colorectal neoplasia

SUPPLEMENTARY MATERIALS

Supplementary Table 1: Clinico-pathological characteristic of patients (n=123) in discovery (n=140) sample sets.

See Supplementary File 1

Supplementary Table 2: MSH3, MSH6 and APC variants in discovery sample sets.

See Supplementary File 2
